# The reliability of cold region tunnels considering deterioration of the insulation layer

**DOI:** 10.1371/journal.pone.0320201

**Published:** 2025-04-04

**Authors:** Jianqing Jia, Shaohua An, Victor O. Tenorio

**Affiliations:** 1 School of Traffic and Transportation, Lanzhou Jiaotong University, Lanzhou, China; 2 Department of Mining and Geological Engineering, University of Arizona, Tucson, Arizona, United States of America; ICFAI Foundation for Higher Education Faculty of Science and Technology, INDIA

## Abstract

Frost heave is a most common form of tunnel defect in cold regions. Installing an insulation layer is a key solution. However, the insulation layer deteriorates with freeze-thaw cycles and temperature changes. These significantly impact tunnel reliability in these areas. To determine the influence of insulation layer deterioration and temperature change to reliability of cold region tunnel, this paper examines a tunnel in the Qinghai-Tibet Plateau. It uses projected temperature increases of 2.6 °C and 4 °C over the next 50 years as boundary conditions. The study analyzes the evolution of freeze-thaw thickness, temperature fields, and stress fields in the surrounding rock. It considers both scenarios: with and without insulation layer deterioration. Using the Monte-Carlo method, the study investigates the variation in tunnel reliability. The results indicate that, with insulation layer deterioration, freeze-thaw thickness, temperature fields, and stress fields in the surrounding rock increase under both temperature scenarios. Consequently, tunnel reliability decreases. Specifically, with temperature increases of 2.6 °C and 4 °C, tunnel reliability decreases by approximately 4.5% and 6.3%, respectively, when considering insulation layer deterioration.

## 1. Introduction

In recent years, with the rapid development of the economy and society, and the implementation of major national strategies such as “One Belt, One Road” and “Transportation Power”, China has constructed numerous transportation infrastructures in high-altitude and low-temperature areas [1]. Due to the topography and geomorphology, many tunnels have been built [2]. Freezing damage is the most common issue in tunnels in cold regions [3]. This damage primarily manifests as water leakage and ice formation on tunnel linings. These issues not only severely damage the engineering structure but also pose significant safety hazards for tunnel operations. Therefore, the effective measures combined with the engineering practice are required to ensure the construction and operation safety of cold region tunnels.

The freeze-thaw cycle is the primary cause of freezing damage in cold region tunnels. When temperatures drop, the moisture in the surrounding rock freezes, expands, and generates frost-heave force [4]. As temperatures rise, the ice melts, leading to volume shrinkage and stress release. Repeated freeze-thaw cycles can easily deform the tunnel lining structure, leading to stress concentration and material fatigue, seriously affecting the tunnel’s structural stability and service life. For a long time, anti-freezing and thermal insulation of cold region tunnels have been significant scientific issues [5–7]. Cui et al. [8] used off-wall thermal insulation lining in a high-speed rail tunnel in cold regions. This significantly improved the freezing damage problem of tunnels. Gao et al. [9] studied the Caomugou Tunnel and proposed an air curtain thermal insulation system. They analyzed its influence on the radial temperature field of surrounding rock using a steady-state heat transfer model. Wang et al. [10] proposed a new thermal insulation technology using composite lining and studied its effectiveness in preventing frost damage. Cui et al. [11] designed an NSCP steel lining and verified its thermal insulation performance through numerical simulation and field experiments. The results can provide a scientific reference for the prevention and control of frost damage in tunnels in the monsoon freezing zone. Chen et al. [12] tested insulation materials in the Dabanshan Tunnel. They optimized the material combination and construction technology of the thermal insulation layer. Ma [13] determined the thickness of the thermal insulation layer on the surface of the secondary lining using numerical simulation. They developed an anti-freeze scheme for a cold region tunnel. Xia et al. [14] optimized the length of the insulation layer in non-permafrost sections using a direct coupling method and proposed anti-frost measures, including improving the frost resistance of concrete, setting cold-proof insulation doors, and installing snow-proof sheds.

In the traditional design of cold region tunnels, thermal insulation is a widely used and effective measure to prevent freezing damage [15]. However, freeze-thaw cycles accelerate the aging of thermal insulation materials. This affects their thermal insulation and antifreeze performance, which is detrimental to the long-term service of tunnels. To address this issue, scholars have conducted extensive experiments and research. Li et al. [16] investigated the effect of mass water content and pore water pressure distribution on the thermal conductivity of tunnel insulation materials after water absorption and immersion. Pei et al. [17] examined the effects of water content, porosity, and solid-phase thermal conductivity on the thermal conductivity of materials. They used three-dimensional model analysis and temperature monitoring comparison. Li et al. [18] analyzed the performance of polyphenol and polyurethane thermal insulation materials under water-rich conditions through on-site monitoring and simulation tests. They concluded that the corrected thickness of the thermal insulation layer should be 0.5 m, considering the influence of air humidity. Han et al. [19] studied the water absorption rate and microscopic characteristics of polyphenolic formaldehyde, polystyrene, and rigid polyurethane thermal insulation materials under water immersion and freeze-thaw cycle conditions. They established a thermal conductivity calculation model. Yang [20] examined the performance of thermal insulation materials under freeze-thaw cycle conditions and found that the microscopic pore structure was destroyed, leading to increased water absorption rates and deterioration of the thermal insulation layer. Tang et al. [21] investigated the durability of polyurethane insulation materials when sandwich laying is adopted. Ding et al. [22] studied the change law and characteristics of thermal conductivity and thermal insulation performance of rigid polyurethane, foam concrete, and vacuum insulation board through freeze-thaw cycle tests. Li et al. [23] analyzed the changes in physical and mechanical properties of thermal insulation materials EPS, PU, XPS, and FLK under freeze-thaw cycles. Wang et al. [24] found that the thermal conductivity of XPS thermal insulation materials increases with the increase of moisture content. To date, numerous studies mainly focus on the effects of different insulation measures or explore the properties of insulation materials under freeze-thaw cycling conditions.

Temperature is the main factor in the research and control of frost damage in cold region tunnels [25]. It significantly impacts the freeze-thaw range, frost heave force, material strength, and long-term structural stability. It may also accelerate the deterioration of thermal insulation materials. Global warming is a key problem that human society needs to face and solve in the future. Qin Dahe et al. [26] believe that the temperature of the Qinghai-Tibet Plateau will rise by about 2.6 °C in the next 50 years. The sixth assessment report of Intergovernmental Panel on Climate Change (IPCC) suggests that, based on estimated global greenhouse gas emissions, the global temperature rise in the 21st century may exceed 1.5 °C. Additionally, the temperature in the third pole is warming at twice the global average rate.

This study aimed to investigate the distributions of temperature and tress fields of a cold region tunnel, with or without considering insulation layer deterioration, at temperature rises of 2.6 °C and 4 °C over the next 50 years. It also calculated the reliability under different conditions. First, a deterioration equation for the insulation layer was developed, and the governing equations for the temperature and stress fields were determined. Second, a numerical simulation model was used to investigate these fields in a cold region tunnel. Finally, the reliabilities of the tunnel under different conditions were calculated using the Monte Carlo method. This study not only considers the impact of insulation layer deterioration on the reliability of tunnels in cold regions, but also takes into account the effects of temperature rises. This paper will be useful in optimizing the insulation layer to meet the target reliability of cold region tunnels.

## 2. Calculation model based on thermo-mechanical coupling

### 2.1. Deterioration equation of insulation layer

Laying an insulation layer is the main measure for passive protection of cold region tunnels. Methods include off-wall, sandwich, double layer, and on-wall [27]. The sandwich method can effectively reduce heat conduction, improve the stability and durability of the tunnel structure, and has a moisture-proof effect, making it widely used in practice. Therefore, the thermal insulation layer in this case study is laid using the sandwich method.

Thermal insulation materials such as polyurethane, polystyrene, and polyphenolic aldehyde can be used in cold region tunnels. Heat transfer mainly depends on the heat conduction of the solid skeleton and internal air. However, during tunnel service, the freeze-thaw cycle can destroy the skeleton structure of the thermal insulation material. It changes its microscopic morphology and causes the internal air medium to be replaced by water or ice with higher thermal conductivity. This leads to the deterioration of the thermal insulation material [28].

Thermal conductivity is an important criterion for evaluating the performance of the thermal insulation layer. The thermal conductivity of thermal insulation materials increases with the number of freeze-thaw cycles. This seriously impacts the antifreeze and thermal insulation effects [29].

Polyurethane thermal insulation materials deteriorate slowly and are widely used in cold region tunnels. Based on the experimental data provided in the literature [29], the governing equation for thermal conductivity of the thermal insulation layer in this study can be expressed as:


λ=0.00021t+0.033 (1)

where λ is the material thermal conductivity(W/(m·°C)) and *t* is the time (year).

### 2.2. Governing equations of temperature field and stress field

The following assumptions are made to develop the governing equations [30,31]: Ignoring material migration. Ignoring the convective heat transfer of tiny soil particles. The tunnel lining is isotropic, and the linear expansion coefficient does not change with time and temperature.

According to the laws of thermodynamics and conservation of energy, the heat conduction equation is as follows [30,31]:


$$\lambda \left(\frac{\partial^{2}T}{\partial x^{2}}+\frac{\partial^{2}T}{\partial y^{2}}+\frac{\partial^{2}T}{\partial z^{2}}\right)+\text{Φ}=\rho c\frac{\partial T}{\partial t}$$ (2)

where Ф is the heat of per unit volume generated by the internal heat source (W/m³), *ρ* is the density (kg/m³); *c* is the specific heat capacity (J/(kg °C)); *T* is the temperature (°C).

When there is a temperature difference in the tunnel lining and it is constrained, the temperature stress can be expressed as [30,31]:


$$v\varepsilon_{x}=\frac{1}{E}[\sigma_{x}-v(\sigma_{y}+\sigma_{z})]+\alpha \Delta T+\varepsilon_{sh}$$ (3)

where ν is the Poisson’s ratio of concrete, ε_x_ is the strain in x-direction, *E* is the elastic modulus (Pa), *σ*_x_, *σ*_y_ and *σ*_z_ are the positive stresses in x, y and z-directions (Pa), respectively, α is the linear expansion coefficient (1/ °C), ∆*T* is the temperature difference (°C), and *ε*_sh_ is the dry shrinkage strain.

### 2.3. Calculation model building

#### 2.3.1. Project overview.

The tunnel in this study is an extra-long highway tunnel located in a high-altitude, cold region. The right line is approximately 5000 meters long, while the left line is approximately 4965 meters long. The elevations at the entrance and exit are 3097 meters and 3068 meters, respectively. The annual average temperature at the tunnel site is − 3.1 °C, with an annual extreme minimum temperature of − 34 °C. The winter period lasts for more than 200 days. The surrounding rock of the tunnel is mainly classified as Grade IV and Grade V.

#### 2.3.2. Numerical model and boundary conditions.

**Fig 1:**
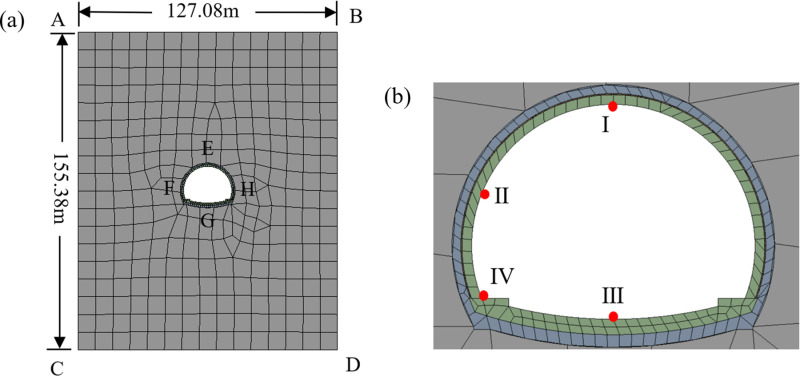
Numerical simulation model. **(a)** Model size, **(b)** The key points.

The ZK84+720 section of the left line, located 100 meters away from the entrance, is selected for numerical simulation in this study. The model is shown in Fig 1.

In the model, both the AC and BD sides are horizontal displacement constraints. The CD side is a fixed boundary, and the AB side is a free boundary. Because the AC and BD sides are far from the tunnel inner wall, temperature changes have less influence on the surrounding rock near the tunnel lining, making them adiabatic boundaries [32,33]. The CD side is the heat flow boundary with a heat flow density of 0.06 W·m². The AB and EFGH sides are the convective heat transfer boundaries with a convective heat transfer coefficient of 15.0 W·m^ −2^.°C^ −1^ [33].

The tunnel temperature was written as [30,34]:


$$T=T_{m}+T_{u}+T_{a}sin\left(\frac{2\times \pi \times time}{365}+\varphi \right)$$ (4)

where *T* is the ambient temperature; *T*_m_ is the annual average temperature; *T*_u_ is the temperature elevated in a certain time; *T*_a_ is the annual temperature amplitude; *time* is the time; *φ* is a phase parameter related to time.

When the temperature increases by 2.6 °C in the next 50 years [26], the temperature boundary conditions of AB side and EFGH side can be written as Equations (5) and (6), respectively.


$$T=-3.1+2.5+\frac{2.6\times time}{50\times 8760\times 3600}+12sin\left[\frac{2\times \pi \times time}{8760\times 3600}+sin^{-1}\left(\frac{6.11}{12}\right)\right]$$ (5)

$$T=-3.1+\frac{2.6\times time}{50\times 8760\times 3600}+12sin\left[\frac{2\times \pi \times time}{8760\times 3600}+sin^{-1}\left(\frac{6.11}{12}\right)\right]$$ (6)

For the same reason, they can be written as Equations (7) and (8), respectively, when the temperature increases by 4 °C in the next 50 years [30,34,35].


$$T=-3.1+2.5+\frac{4\times time}{50\times 8760\times 3600}+12sin\left[\frac{2\times \pi \times time}{8760\times 3600}+sin^{-1}\left(\frac{6.11}{12}\right)\right]$$ (7)

$$T=-3.1+\frac{4\times time}{50\times 8760\times 3600}+12sin\left[\frac{2\times \pi \times time}{8760\times 3600}+sin^{-1}\left(\frac{6.11}{12}\right)\right]$$ (8)

In this study, the thermodynamic parameters of the materials [33] are shown in Table 1.

**Table 1:** Thermodynamic parameters.

Materials		Density (kg·m^ −3^)	Thermal conductivity (W·m^ − 1^°C^ −1^)	Specific heat capacity (J·kg^ − 1^°C^ −1^)
Surrounding rock	Unfrozen	1850	1.982	1876
	Frozen	1800	2.5	869
Primary support		2500	1.38	989
Secondary lining		2500	1.47	1106
Insulation layer		41	0.033	1756

## 3. Calculation results and discussions

The temperature increase significantly impacts the temperature field, stress field, and freeze-thaw range of tunnel surrounding rock in cold regions. During the freeze-thaw cycle, the surrounding rock and support structure deteriorate to a certain extent. This deterioration reduces the stability of the tunnel. Therefore, temperature boundary conditions are a key factor in the stability calculation of cold region tunnels. Based on the latest research results, the practical study in this section only considers two temperature boundary conditions: 2.6 °C and 4 °C.

### 3.1. Temperature field with 2.6 °C boundary condition

#### 3.1.1. Without considering insulation layer deterioration.

**Fig 2:**
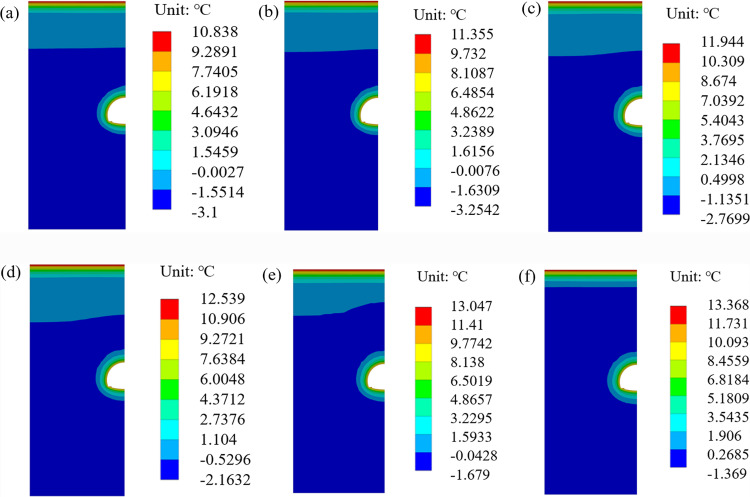
Temperature field without considering insulation layer deterioration (2.6 °C). **(a)** 0a, **(b)** 10a, **(c)** 20a, **(d)** 30a, **(e)** 40a, **(f)** 50a.

The temperature field without considering the deterioration of the insulation layer is shown in Fig 2.

Fig 2 shows that the surrounding rock near the inverted arch does not thaw seasonally in the first 20 years without insulation deterioration. As the temperature increases, the maximum freeze-thaw range of the surrounding rock [36] in the 30th, 40th, and 50th years is 0.12 m, 0.16 m, and 0.18 m, respectively. The latter increased by 33% and 13%, respectively, compared to the former.

In the 10th year, the temperature of the inverted arch is 8.86 °C, which is 6.2% higher than the initial temperature of 8.34 °C, showing the same increasing trend as the air temperature [37]. In the 20th, 30th, 40th, and 50th years, the maximum temperature of the surrounding rock is 9.92 °C, 10.52 °C, 11.02 °C, and 11.34 °C, respectively. The growth rate of each decade is 6.3%, 5.8%, 4.8%, and 2.7%, respectively, indicating that the growth rate of the maximum temperature gradually decreases after 30 years.

#### 3.1.2. Considering insulation layer deterioration.

**Fig 3:**
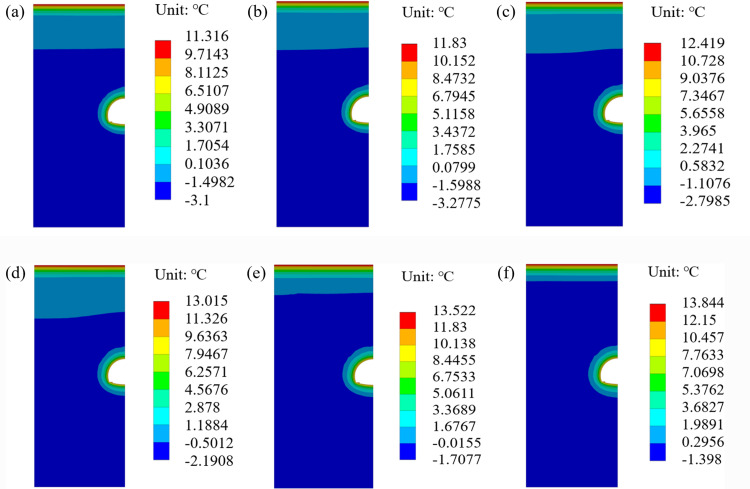
Temperature field considering the insulation layer deterioration (2.6 °C). **(a)** 0a, **(b)** 10a, **(c)** 20a, **(d)** 30a, **(e)** 40a, **(f)** 50a.

**Fig 4:**
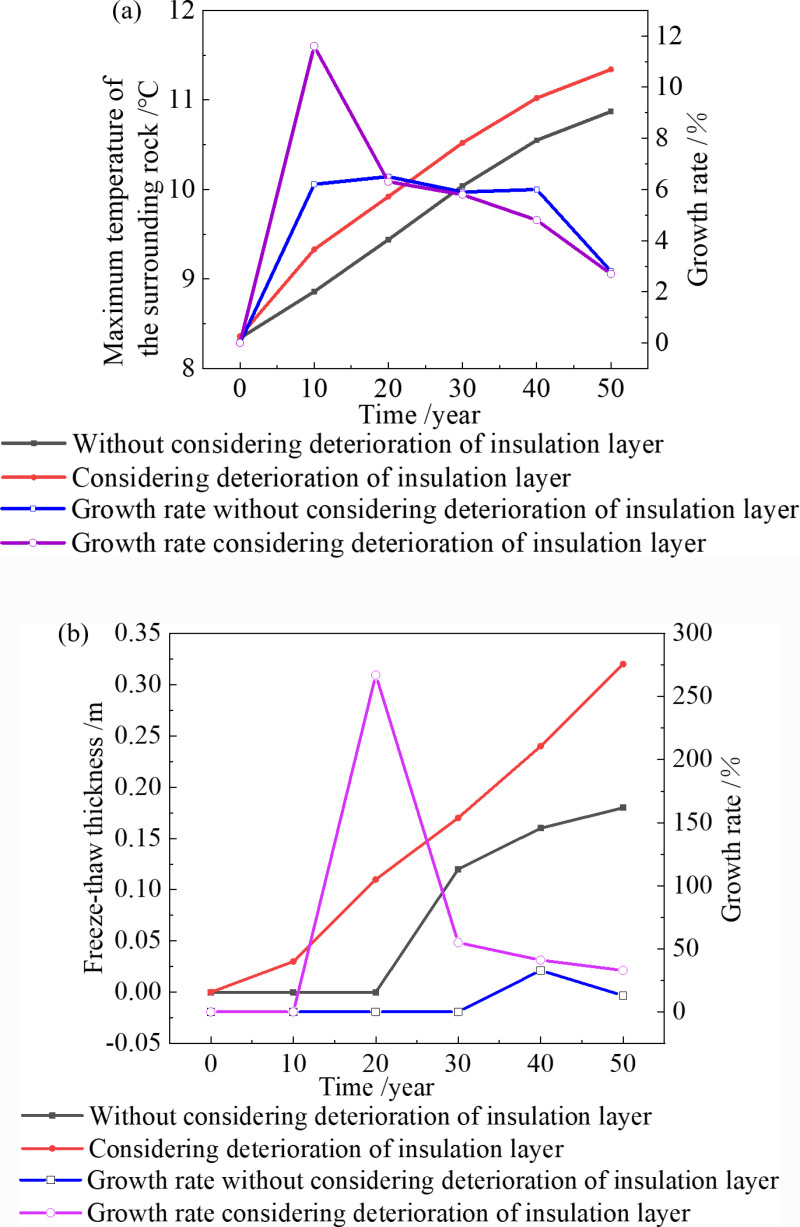
Maximum temperature and freeze-thaw thickness of surrounding rock (2.6 °C). **(a)** Maximum temperature of surrounding rock, **(b)** Freez- thaw thickness of surrounding rock.

The temperature field considering deterioration of the insulation layer is shown in Fig 3. The freeze-thaw range and maximum temperature of surrounding rock are shown in Fig 4. It can be seen from the figures that:

The surrounding rock on both sides of the tunnel has thawed seasonally in the 10th year, and the thickness of the freeze-thaw circle is 0.03 m. The thickness of the freeze-thaw circle [38] in the 20th, 30th, 40th, and 50th years is 0.11 m, 0.17 m, 0.24 m, and 0.32 m, respectively. The thickness increased by 41.2% in the 40th year compared to the 30th year. It increased by 33% in the 50th year compared to the 40th year, while the temperature gradient and growth rate decreased. In the 10th year, the maximum temperature of the surrounding rock near the inverted arch is 9.33 °C, which is 11.6% higher than the initial 8.36 °C. In the 20th, 30th, 40th, and 50th years, the maximum temperature of the tunnel surrounding rock is 9.92 °C, 10.52 °C, 11.02 °C, and 11.34 °C, respectively. The growth rates per 10 years are 6.3%, 5.8%, 4.8%, and 2.7%, respectively. The maximum temperature growth rate of the tunnel surrounding rock gradually decreases after the 30th year.

Fig 4 also shows that when the temperature increases by 2.6 °C over the next 50 years, the freeze-thaw range and temperature of the surrounding rock increase over time. However, the growth rate gradually slows down [39]. When considering the deterioration of the insulation layer, the freeze-thaw range and temperature of surrounding rock are higher than without considering the deterioration. At the 30th, 40th, and 50th years, the freeze-thaw ranges are 0.12 m and 0.17 m (an increase of 42%), 0.16 m and 0.24 m (an increase of 50%), 0.18 m and 0.32 m (an increase of 78%), respectively. The temperatures are 10.04 °C and 10.52 °C (an increase of 4.8%), 10.55 °C and 11.02 °C (an increase of 4.5%), 10.87 °C and 11.34 °C (an increase of 4.3%).

### 3.2. Temperature field with 4 °C boundary condition

**Fig 5:**
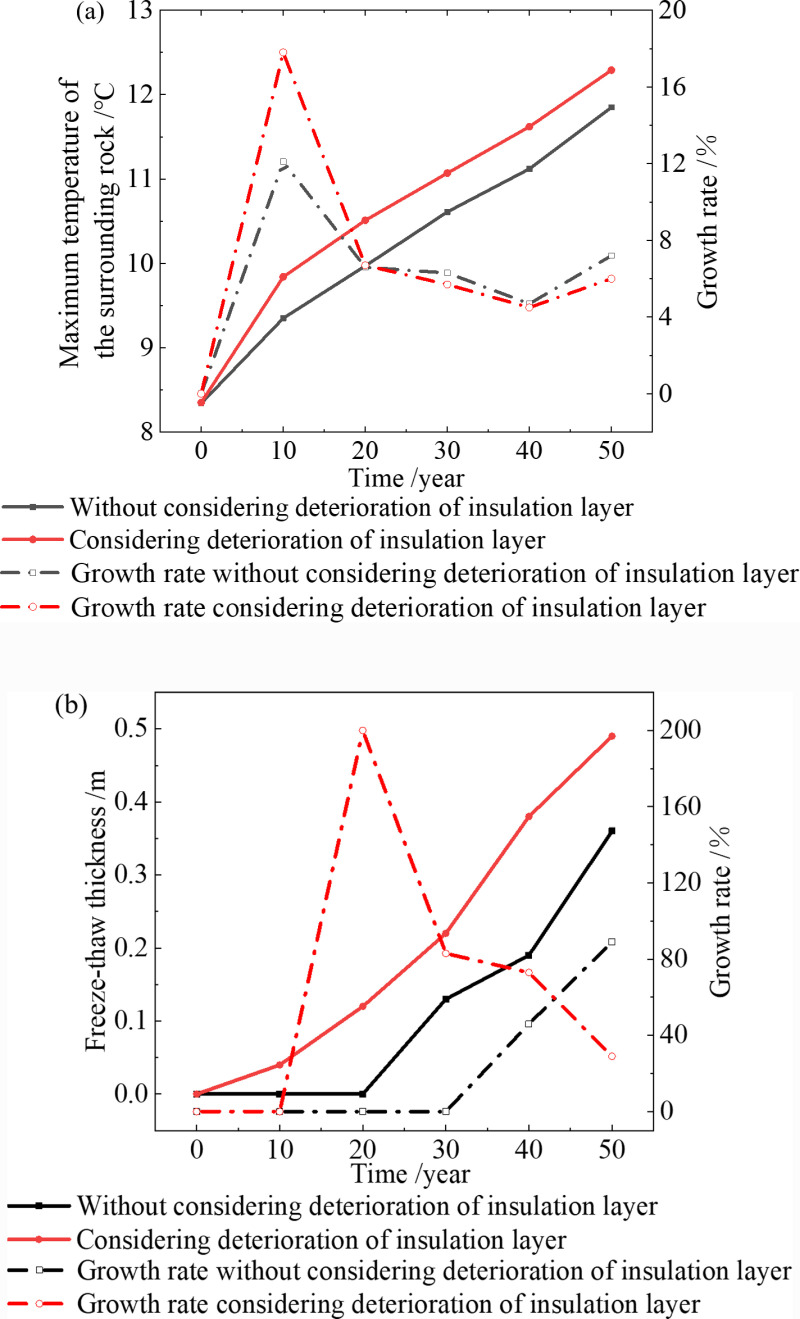
Maximum temperature and freeze-thaw thickness of surrounding rock (4 °C). **(a)** Maximum temperature of surrounding rock, **(b)** Freeze-thaw thickness of surrounding rock.

When the temperature increases by 4 °C in the next 50 years, the thickness of freeze-thaw circle and the maximum temperature of the surrounding rock are shown in Fig 5.

Here are the simulation results for the maximum temperature and freeze-thaw thickness of the surrounding rock, as depicted in Fig 5:

The change in the freeze-thaw range and maximum temperature is similar to that of a 2.6 °C increase when the temperature increases by 4.0 °C, but both the thickness of the freeze-thaw circle and the maximum temperature of the surrounding rock increase. When the deterioration of the insulation layer is not considered, the thickness of the freeze-thaw circle in the 50th year is 0.36 m, which is 0.18 m higher than that at 2.6 °C, and the highest temperature of the surrounding rock is 11.85 °C, which is 0.98 °C higher than that at 2.6 °C. When the deterioration of the insulation layer is considered, the thickness of the freeze-thaw circle in the 50th year is 0.49 m, which is 0.17 m higher than that at 2.6 °C, and the highest temperature of the surrounding rock is 12.29 °C, which is 0.95 °C higher than that at 2.6 °C. In the 50th year, the thickness of freeze-thaw circle and maximum temperature of the surrounding rock, considering the deterioration of the insulation layer, increased by 36% and 4%, respectively, compared to those without considering the deterioration of the insulation layer.

### 3.3. Stress field with 2.6 °C boundary condition

#### 3.3.1. Without considering insulation layer deterioration.

**Fig 6:**
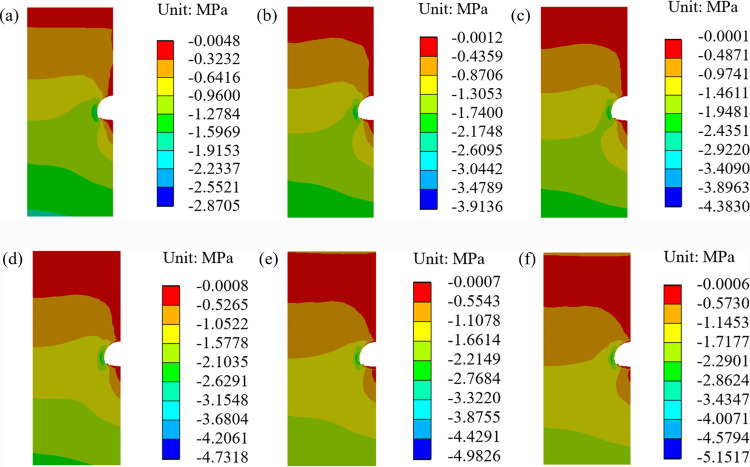
Stress field of surrounding rock without considering insulation layer deterioration (2.6 °C). **(a)** 0a, **(b)** 10a, **(c)** 20a, **(d)** 30a, **(e)** 40a, **(f)** 50a.

**Fig 7:**
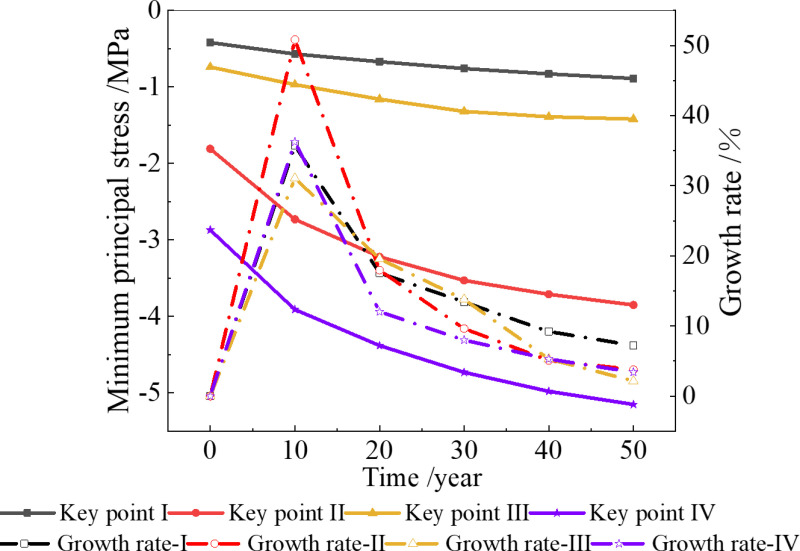
Minimum principal stress without considering the insulation layer deterioration (2.6 °C).

When the temperature increases by 2.6 °C over the next 50 years, the stress field and minimum principal stress, without considering the deterioration of the insulation layer, are shown in Figs 6 and 7.

As seen from Figs 6 and 7, the stress of the surrounding rock increases over time, but the growth rate gradually slows down [40]. The initial stress is the largest at point IV, which is 2.87 MPa. In the 10th year, the stresses at points I, II, III, and IV are 0.57 MPa, 2.73 MPa, 0.97 MPa, and 3.91 MPa, respectively. These represent increases of 36%, 51%, 31%, and 36% compared to the initial stress. In the 20th, 30th, 40th, and 50th years, the maximum stresses are 4.38 MPa, 4.73 MPa, 4.98 MPa, and 5.15 MPa, respectively.

#### 3.3.2. Considering insulation layer deterioration.

**Fig 8:**
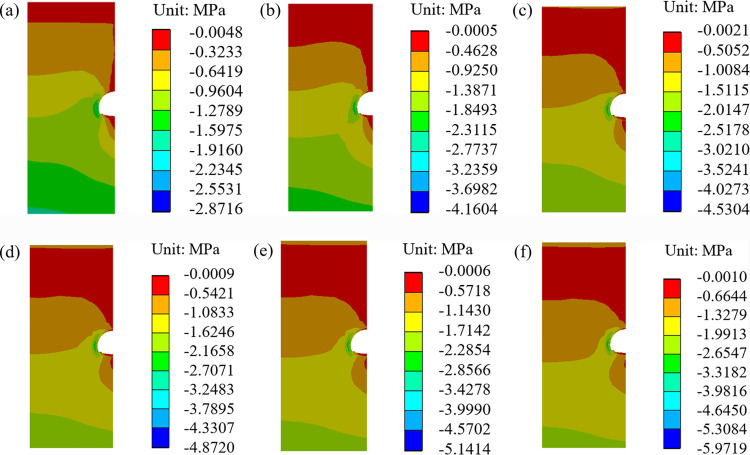
Stress field of surrounding rock considering the insulation layer deterioration (2.6 °C). **(a)** 0a, **(b)** 10a, **(c)** 20a, **(d)** 30a, **(e)** 40a, **(f)** 50a.

**Fig 9:**
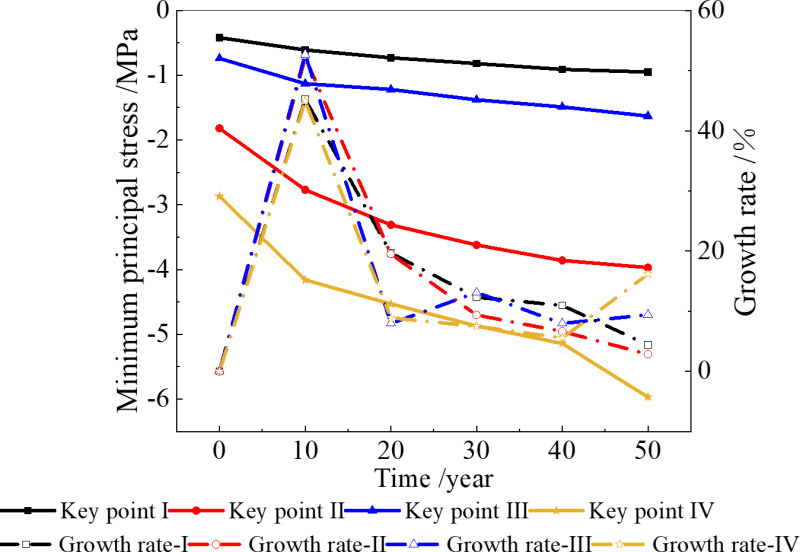
Minimum principal stress considering the insulation layer deterioration (2.6 °C).

When the temperature increases by 2.6 °C over the next 50 years, the stress field distribution and minimum principal stress of the surrounding rock, considering the deterioration of the insulation layer, are shown in Figs 8 and 9.

It can be seen from Figs 8 and 9 that the stress of the surrounding rock also increases with the passage of time, but the growth rate gradually decreases [40–42]. In the 10th year, the stresses at key points I, II, III, and IV are 0.61 MPa, 2.77 MPa, 1.13 MPa, and 4.16 MPa, representing increases of 45%, 52%, 53%, and 45%, respectively, compared with the initial stress. In the 20th, 30th, 40th and 50th years, the maximum compressive stresses of the surrounding rock of the tunnel are 4.53 MPa, 4.87 MPa, 5.14 MPa, and 5.97 MPa.

From the above results, after considering the deterioration of the insulation layer, the stress at the four key points in the same period will increase compared to that without considering the deterioration of the insulation layer. The largest increase is 0.82 MPa, which occurs in the 50th year. To ensure long-term stability, it is necessary to consider the deterioration of the insulation layer.

### 3.4. Stress field with 4 °C boundary condition

**Fig 10:**
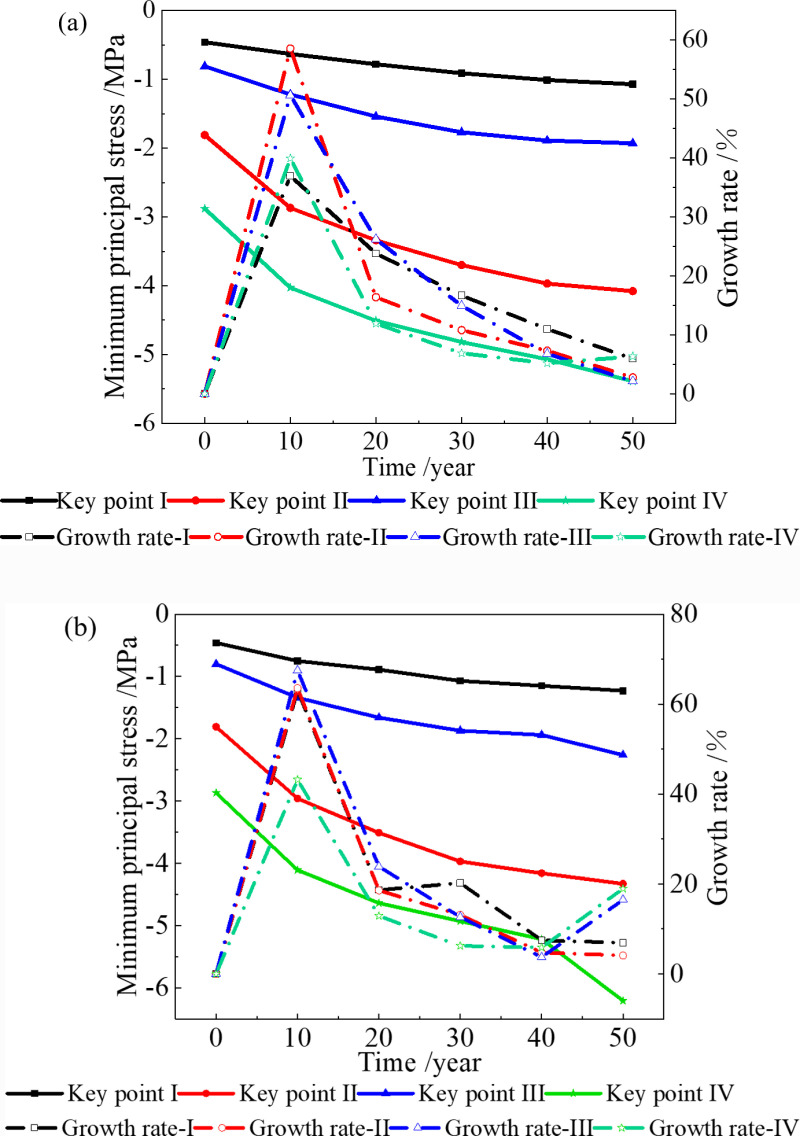
Minimum principal stress of surrounding rock when the temperature increases by 4 °C. **(a)** Without considering the deterioration of insulation layer, **(b)** Considering the deterioration of insulation layer.

When the temperature increase 4 °C in the next 50 years, the minimum principal stress changes at the four key points are shown in Fig 10.

Fig 10 shows that when the temperature increases by 4 °C over the next 50 years, the compressive stress at each key point increases compared to that at 2.6 °C. Without considering the deterioration of the insulation layer, the maximum compressive stress after 50 years is 5.39 MPa, which is 5% higher than that at 2.6 °C in the same period. When considering the deterioration of the insulation layer, the maximum compressive stress after 50 years is 6.21 MPa, which is 4% higher than that at 2.6 °C in the same period.

## 4. Reliability of cold region tunnel considering insulation layer deterioration

**Fig 11:**
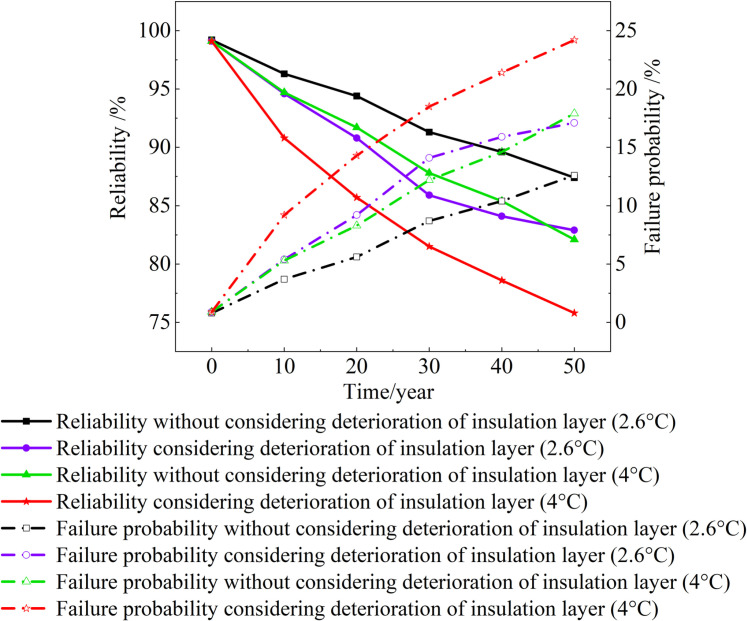
Reliability and failure probability.

In this study, Young’s modulus, density and Poisson’s ratio of the surrounding rock, primary lining, and secondary lining are set as random input variables. The minimum principal stress of tunnel support is regarded as a random output variable. The Monte Carlo method is used to calculate the variation characteristics of tunnel reliability [34,43]. The calculated results are shown in Fig 11.

Fig 11 provides the valuable insights: The failure probability of tunnel under the four conditions gradually increases, and the reliability of tunnels gradually decreases. Under the same temperature condition, the failure probability considering the deterioration of the insulation layer is greater than that without considering the deterioration of the insulation layer. When the temperature increases by 2.6 °C and the deterioration of insulation layer is considered, in the same period, the failure probability of the tunnel is greater than that of 4 °C and without considering the deterioration of the insulation layer. In the 30th year, the tunnel reliability under the four conditions is 91.3%, 85.9%, 87.8% and 81.5%, which decreased by 7.9%, 13.2%, 11.3% and 17.6% compared with the 0th year. In the 50th year, it is 87.4%, 82.9%, 82.1% and 75.8%, respectively.

## 5. Conclusions

In this study, the governing equation and temperature equations for two types of temperature rises are derived. Based on this, with or without considering the insulation layer deterioration, the numerical model is built. The temperature field, freeze-thaw thickness, maximum temperature, maximum stress, and the reliability of the cold region tunnel are all simulated and analyzed for temperature increases of 2.6 °C and 4 °C over the next 50 years. The key findings are given below:

The thickness of the freeze-thaw circle of the tunnel surrounding rock gradually increases over time, with the growth rate decreasing after 40 years. When the temperature increases by 2.6 °C, the thicknesses of the freeze-thaw circles in the 50th year are 0.18 m without considering the deterioration of insulation layer and 0.32 m with considering the deterioration. When the temperature increases by 4 °C over the next 50 years, the thicknesses are 0.36 m and 0.49 m, respectively, and it is greater than that of 2.6 °C.

After considering the deterioration of the insulation layer, the thickness of the freeze-thaw circle, maximum temperature, and maximum stress of tunnel surrounding rock will increase compared to those without considering the deterioration. When the temperature increases by 2.6 °C, they increase by 0.14 m, 0.47 °C, and 0.82 MPa. When the temperature increases 4 °C, they increase by 0.13 m, 0.44 °C, and 0.82 MPa. The deterioration of the insulation layer impacts the long term stability of cold region tunnels. Considering the deterioration of the insulation layer, the tunnel reliability decreases by 4.5% and 6.3% over 50 years with temperature increases of 2.6 °C and 4 °C, respectively.

Considering two kinds of temperature boundaries and insulation layer deterioration, the study examined the characteristics of the temperature field, freeze-thaw range, stress field, and reliability, leading to meaningful conclusions. However, important factors and their coupling effects, such as extreme temperature, earthquakes, rainfall, and the geological environment, significantly impact the long-term stability of tunnels in cold regions. This remains an important topic for future research in this field.
